# Development of a specimen-specific in vitro pre-clinical simulation model of the human cadaveric knee with appropriate soft tissue constraints

**DOI:** 10.1371/journal.pone.0238785

**Published:** 2020-10-14

**Authors:** Aiqin Liu, William J. Sanderson, Eileen Ingham, John Fisher, Louise M. Jennings

**Affiliations:** 1 Institute of Medical and Biological Engineering, School of Mechanical Engineering, Faculty of Engineering and Physical Sciences, University of Leeds, Leeds, United Kingdom; 2 Institute of Medical and Biological Engineering, School of Biomedical Sciences, Faculty of Biological Sciences, University of Leeds, Leeds, United Kingdom; University of Memphis, UNITED STATES

## Abstract

A human cadaveric specimen-specific knee model with appropriate soft tissue constraints was developed to appropriately simulate the biomechanical environment in the human knee, in order to pre-clinically evaluate the biomechanical and tribological performance of soft tissue interventions. Four human cadaveric knees were studied in a natural knee simulator under force control conditions in the anterior posterior (AP) and tibial rotation (TR) axes, using virtual springs to replicate the function of soft tissues. The most appropriate spring constraints for each knee were determined by comparing the kinematic outputs in terms of AP displacement and TR angle of the human knee with all the soft tissues intact, to the same knee with all the soft tissues resected and replaced with virtual spring constraints (spring rate and free length/degree). The virtual spring conditions that showed the least difference in the AP displacement and TR angle outputs compared to the intact knee were considered to be the most appropriate spring conditions for each knee. The resulting AP displacement and TR angle profiles under the appropriate virtual spring conditions all showed similar shapes to the individual intact knee for each donor. This indicated that the application of the combination of virtual AP and TR springs with appropriate free lengths/degrees was successful in simulating the natural human knee soft tissue function. Each human knee joint had different kinematics as a result of variations in anatomy and soft tissue laxity. The most appropriate AP spring rate for the four human knees varied from 20 to 55 N/mm and the TR spring rate varied from 0.3 to 1.0 Nm/°. Consequently, the most appropriate spring condition for each knee was unique and required specific combinations of spring rate and free length/degree in each of the two axes.

## Introduction

Knee osteoarthritis affects 4.71 million people in the UK and this number is expected to double by 2035 due to an ageing and increasingly obese population [1]. There is an increasing clinical need for effective early-stage surgical interventions, such as cartilage repair therapies and meniscal repair interventions, which replace or regenerate damaged or diseased soft tissue structures in the knee and therefore prevent or delay the disease process [2–4]. There are, however, no standard pre-clinical test methods to assess the functional performance of these early-stage interventions that can represent the biomechanical environment *in vivo* and also consider variations across patient groups [5].

We previously developed a novel pre-clinical simulation model of the natural whole porcine knee joint, which has been successfully applied to the assessment of the tribological performance of osteochondral grafts in the knee joint [6–8]. The refined porcine knee model [8] was shown to successfully simulate the natural porcine knee ligament function by constraining the anterior posterior (AP) motion using physical compressive springs. The results also highlighted the influence of input parameters of spring constraints (spring rate and free length) on the outputs of the natural porcine knee model including knee kinematics and tribological function. For the porcine model, which had low biological variability due to consistency in key parameters such as knee dimensions and anatomy due to sourcing tissue from pigs of the same breed, age and sex, the kinematic outputs across replicates showed low variability. The porcine model is, therefore, not a specimen-specific model and average spring conditions enabled the simulation of ligament function for all samples studied.

This approach, of simulating the average soft tissue tensions may not, however, be appropriate for natural human knee joints. It is likely that each individual natural human knee joint will behave differently biomechanically, with different knee kinematics as a result of variations in anatomy, soft tissue laxity and associated levels of disease such as osteoarthritis. Therefore, the appropriate soft tissue constraints for the natural human knee joint may require investigation for each individual joint specimen to represent the biomechanical environment *in vivo*. The findings from the porcine knee model provided important guidelines for selecting appropriate soft tissue constraints in the natural knee model with the aim of matching these constraints more closely to the constraints of the soft tissue in individual joints. The porcine knee model also provided a baseline for developing a natural human knee model with appropriate spring constraints to simulate whole soft tissue function.

Soft tissue constraints have been shown to significantly affect the kinematics of knees in experimental simulations [9–12]. Springs have been widely used to simulate soft tissue functions in both computational [13–15] and experimental studies [10, 11, 16–19]. Historically, physical spring constraints were initially used in artificial knee simulators to replicate the soft tissue or ligament function in AP translation and tibial rotation of the natural human knee [16–18, 20]. Linear compression springs were previously used in force-controlled knee simulators, which have been gradually replaced by non-linear springs [21–23] to simulate the toe region of the ligament load-displacement curve identified by Fukubayashi et al. [20]. Van Houtem et al. [17] applied a gap of 2.5 mm in the AP direction between the spring and the tibia in a force-controlled knee simulator to simulate the non-linear toe region of the ligaments. The ISO standard [24] for wear testing of TKR using force control parameters recommends the use of springs with a ± 2.5 mm gap set at the neutral position in the AP direction of the simulator and a gap of ± 6° in the TR restraint system to simulate the laxity of the natural human knee ligament.

Although physical springs can provide repeatable controlled soft tissue constraints for the evaluation of knee mechanics, they can be costly and time-intensive to set up. They also have limited options of spring stiffnesses and gaps, and a limited ability to simulate nonlinear force-displacement characteristics of soft tissue. These reasons have motivated the development of virtual spring constraint systems in experimental knee simulations [10, 11, 19]. These virtual spring constraints in the knee simulator can be programmed to simulate different levels of soft tissue constraint more accurately, and the adjustment of load/torque-displacement/rotation curves are much simpler and time-efficient than the adjustment of physical springs.

Our previous study showed that soft tissue variations affect the knee kinematics and tribological function of the natural porcine knee. This indicated that the soft tissue constraints would also affect the functional performance of early-stage surgical interventions, such as cartilage and meniscal repair and replacement therapies in the natural knee model [8]. Therefore, it is important to develop a method to replicate individual variations in soft tissue constraints in the natural knee simulator in order to more appropriately simulate the biomechanical environment *in vivo*. Such a method is necessary to efficiently and reliably pre-clinically assess the functional performance of early interventions in the knee.

The aim of this study was to develop a human cadaveric knee model with appropriate soft tissue constraints using a virtual spring system. For future applications, for example, to evaluate the functional performance of a cartilage repair technique, it will be desirable to remove the soft tissue and replace it with a virtual spring system to replicate the soft tissue function. As a first step, a human cadaveric knee experimental simulation model with appropriate soft tissue constraints was developed.

For the porcine knee model, one set of AP physical springs in the sagittal plane was used to simulate the primary function of the cruciate ligaments in controlling AP motion. A displacement control profile was applied to the porcine knee model in order to control tibial motion while investigating appropriate constraints in the AP direction [8]. For the human knee model, both the sagittal and transverse planes were investigated with the aim of simulating the total soft tissue function in the knee joint. The human knee model used a force control profile to drive both AP force and tibial rotation (TR) torque in order to simulate soft tissue function under external force. The kinematic outputs in terms of AP displacement in the sagittal plane, and TR angle in the transverse plane, of the human knee with soft tissues removed and constrained with different virtual spring conditions were compared with those of the natural human knee with all of the soft tissues intact. The spring conditions that showed the least difference in AP displacement and TR angle output compared to the intact knee were considered as the most appropriate spring conditions for each individual knee.

## Materials and methods

### Human knee joint

The human knee simulation model was developed using a single station natural knee joint simulator (Simulation Solutions, Stockport, UK), which has been described previously [6–8].

**Fig 1:**
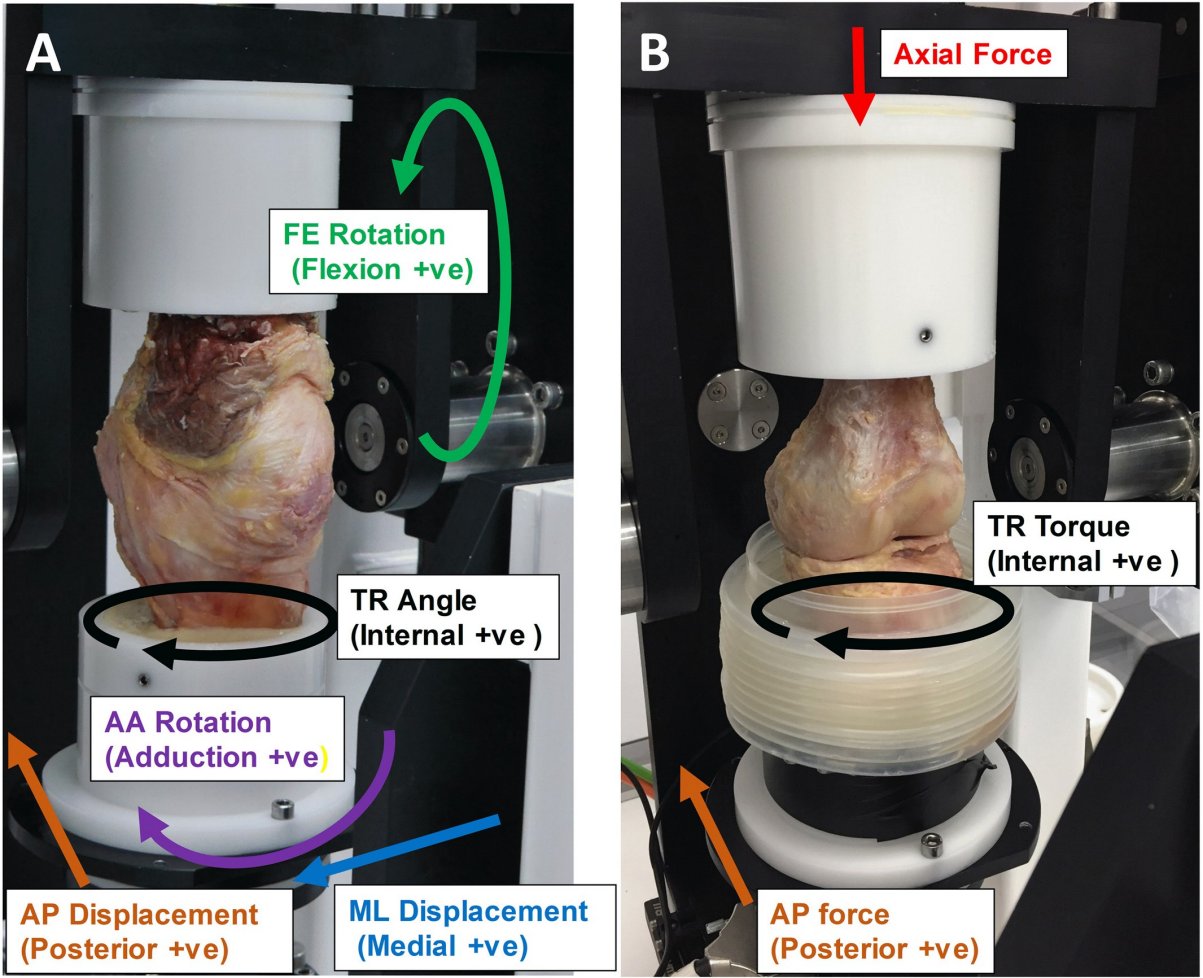
A) Intact human left cadaveric knee specimen and B) dissected human left cadaveric knee specimen set up in the single station knee simulator with indications of A) kinematic inputs and outputs and B) force inputs.

The study was approved by East Midlands—Leicester South Research Ethics Committee (UK) with an approval number of 18/EM/0224. Human cadaveric knees were imaged using magnetic resonance imaging (MRI) (Siemens Magnetom Prisma (3T), Erlangen, Germany) to eliminate specimens with any prior trauma, ligament or meniscus injury, malalignment, or deformity. Four cadaveric knees (age range 47–76 years old, mean age 62 years, one female and three male, no history of previous knee surgery) were selected and studied in the single station knee simulator. For each knee, for the first part of the study, the knee joint capsule including synovial fluid and suprapatellar pouch were kept intact, including all soft tissue. The proximal femur and distal tibia were cleaned of soft tissues to enable placement of the femur and tibia into cement pots (Fig 1). The knee was cemented using polymethylmethacrylate (PMMA; WHWPlastics, UK) and mounted to the knee simulator for investigation according to our previous study [6]. Throughout the procedure, the soft tissues were kept moist using Ringer’s solution (Sigma-Aldrich, Buchs, Switzerland). The axial force axis was shifted medially by 0.07 of the tibial width by sliding the tibial pot in the fixture with the aim of causing greater medial compartment loading [6].

### Intact human knee study (soft tissue retained, control)

**Fig 2:**
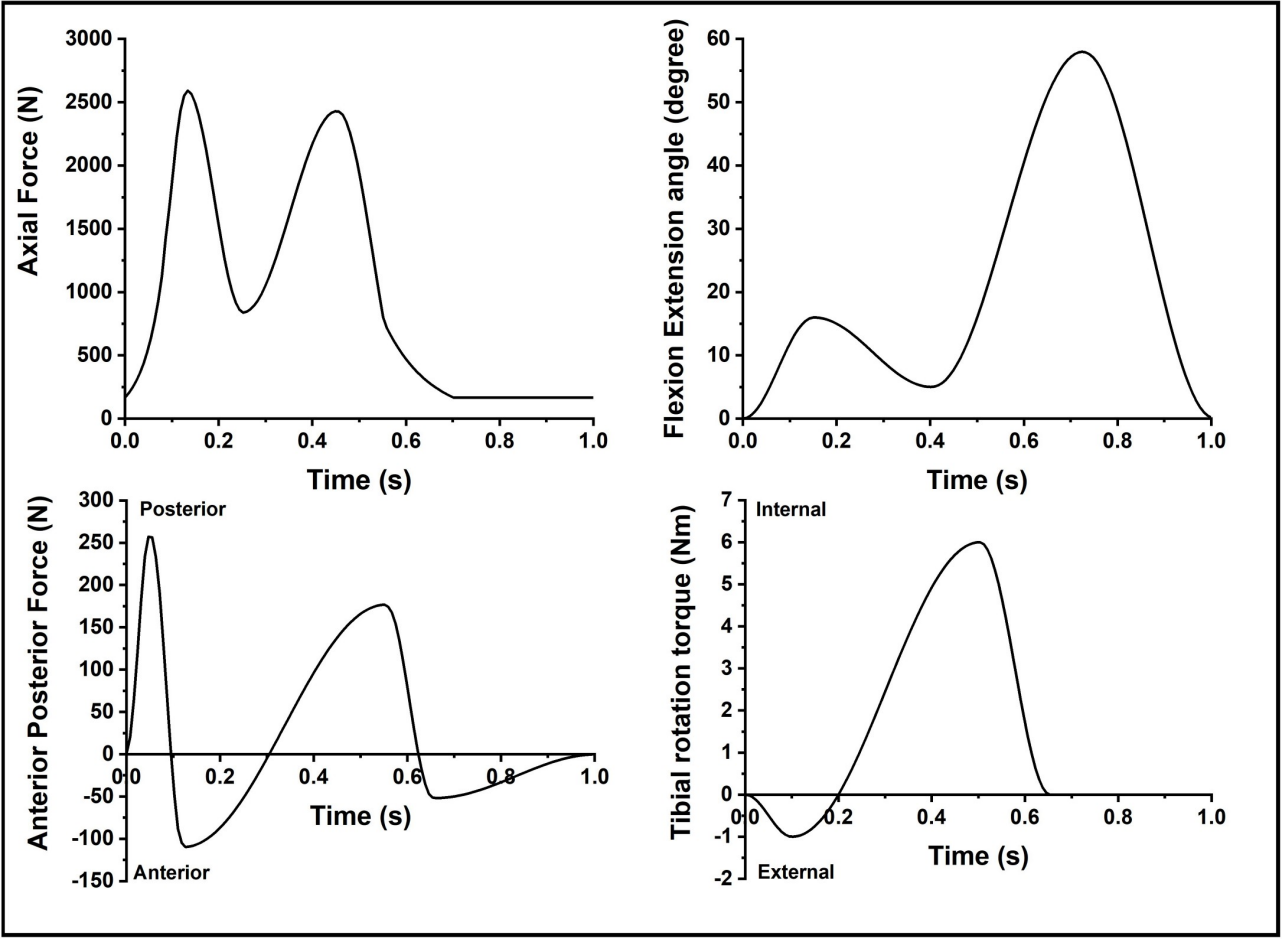
Kinematic input profiles for the human cadaveric knee (derived from [24]).

A human knee walking gait cycle profile, modified from the international standard for wear of total knee-joint prostheses using force control (ISO14243-1-2009) [24], was applied as shown in Fig 2. The use of force control for AP and TR for this study allowed the different soft tissue constraints, represented by the springs, to control the output kinematics of the joint. The force and torque waveforms from ISO14243-1-2009 [24] are based on healthy subject data from the study of Morrison et al. [25]. With the development of measurement technology, more recent studies have revealed only two peaks in axial force instead of the three peaks from the ISO standard [26, 27]. Therefore, a modified ISO axial force profile with two peaks (Fig 2) was applied in this study in order to more closely simulate the natural knee. The flexion/extension (FE), AP force and TR torque were adopted from the ISO force control standard (Fig 2).

**Fig 3:**
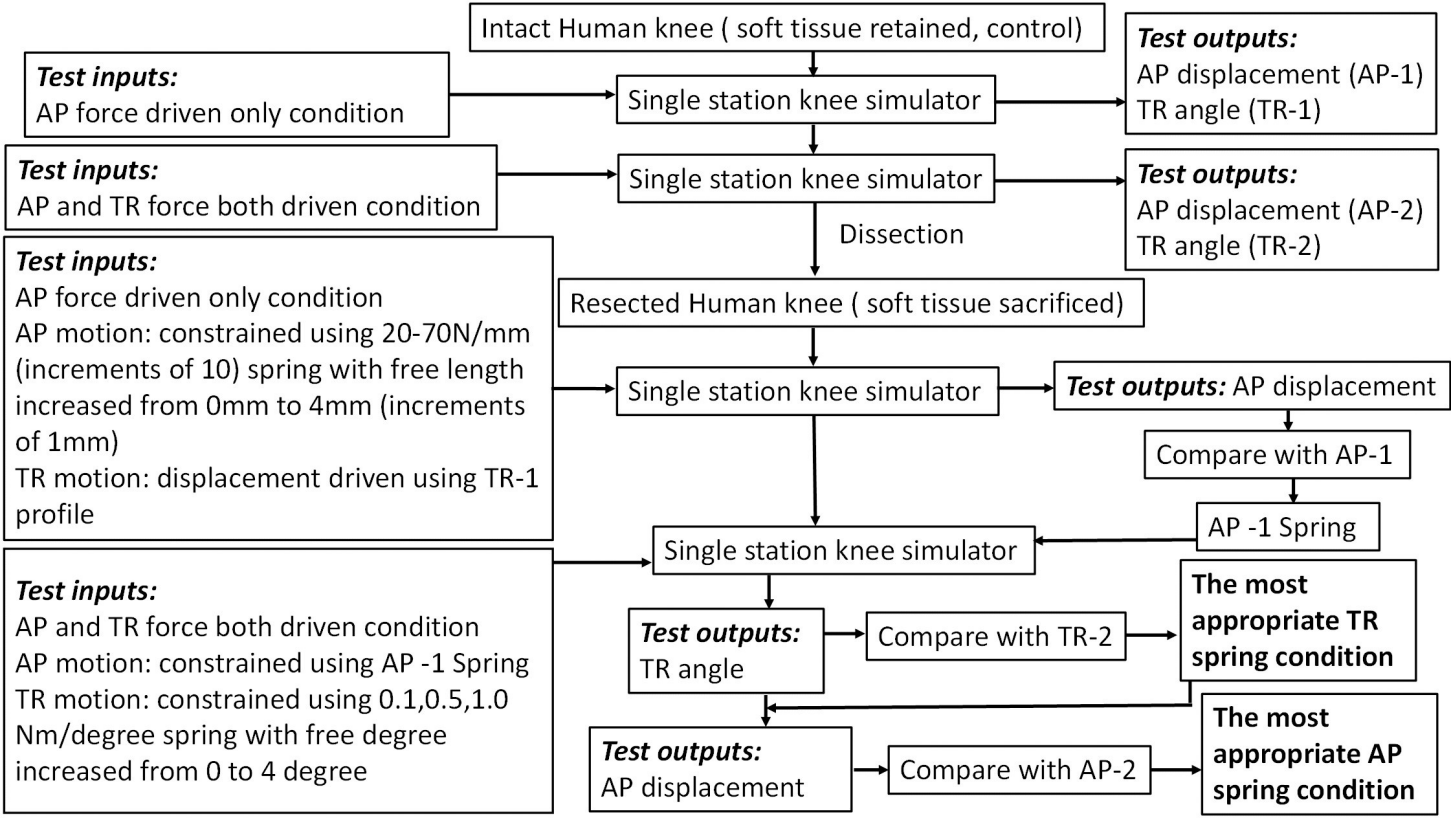
Flow chart of the study method for each human knee specimen.

The detailed test procedure for each individual knee is shown in Fig 3. The intact knee was initially studied under ‘AP force driven only’ condition, in which the AP motion was force driven while the TR was allowed to move freely. The output kinematics including AP displacement (AP-1) and TR angle (TR-1) were recorded under this condition, which was then used to investigate the AP spring conditions for the resected human knee while the TR was displacement controlled using the TR output (TR-1) profile obtained from the intact knee as the TR input. The same intact knee was then studied under ‘AP and TR force both driven’ condition, in which both the AP and TR motion were force driven. The output kinematics including AP displacement (AP-2) and TR angle (TR-2) were recorded under this condition, which was used to investigate both AP spring and TR spring conditions for the resected knee. This two-stage approach was undertaken since there were two unknown spring constraints in the AP and TR directions to be investigated. Therefore, determining the constraints in two steps rather than attempting to determine the constraints simultaneously reduced the risk of instability and dislocation. Each study was run for 10 cycles at 1Hz after the test was stabilised, and consistent data was generated. The abduction/adduction (AA) motion was left unconstrained while the medial/lateral (M/L) displacement was constrained in all instances as previously stated.

### Resected human knee study (soft tissue sacrificed)

All the soft tissues for each knee were then sacrificed leaving only the meniscus and cartilage in place. The knee was mounted in the simulator with Ringer’s solution as the lubricant. Virtual AP springs and TR springs with different spring rates and free lengths/degrees were used to simulate soft tissue function by constraining the AP and TR motion according to our previous study [10]. Virtual spring profiles with different spring rates and free lengths/degrees were programmed and uploaded to the simulator.

Tuning of the simulator was required at the beginning of each test in order to make sure the force outputs achieved the demand force accurately. Preliminary studies showed that a free length larger than 4 mm for AP springs and a free rotation larger than 4° for the TR springs caused difficulties in the tuning of the AP and TR forces respectively in the simulation. The force tuning of the simulator was highly sensitive to the stability of the contact surface of the cadaveric sample. Therefore, a sample that had free motion greater than the above threshold values had a higher risk of dislocation of the femoral-tibial joint, causing knee instability and eventually damage of the cadaveric knee contact surfaces. As suggested by our previous porcine knee model [8], in addition to spring stiffness and free length, the stability of the knee needs to be taken into account in selecting appropriate spring constraint conditions for the simulation of the natural knee joint. Therefore, in order to maintain the stability of the knee joint and enable the measured forces to closely follow the demand force profile, only spring constraints with gaps less than 4 mm or 4° were applied in this study. Using these threshold values there was no separation or dislocation of the femur and tibia observed in all of the four cadaveric knees in any spring conditions applied in this study.

Our previous study [8] demonstrated that either increasing the free length/rotation or decreasing the spring rate would increase the AP displacement or TR angle. Therefore, for knee samples which needed larger than 4 mm or 4° to match the profiles of AP displacement or TR angle, a reduced spring rate was applied rather than increasing the free length /rotation gap.

Initially, the knee was studied under the condition ‘AP force driven only’ and the level of AP constraint was controlled by the virtual AP springs with different spring rates and free lengths in both the anterior and posterior directions (Fig 3). The TR motion was driven in displacement control by applying the TR angle output profile (TR-1) from the intact knee study. Each spring condition was run for 10 cycles at 1Hz and the AP displacement was measured. The most appropriate AP spring condition under the AP force driven only condition (AP-1 spring) was determined first, by comparing the AP displacement outputs at different AP spring conditions with those of the intact knee for each knee (AP-1). The knee was then studied under the ‘AP and TR force both driven’ condition by applying the AP-1 spring in the AP direction and different TR springs in the TR direction (Fig 3). The most appropriate TR spring condition was selected by comparing the TR angle outputs at these different TR spring conditions with those of the intact knee for each knee (TR-2). In the final stage, the initially selected AP spring condition (AP-1 spring) was further adjusted under this ‘AP and TR force both driven’ condition to match the AP displacement output (AP-2), whilst the most appropriate TR spring condition was simultaneously applied in the TR direction.

Three time ranges were chosen for analysis of AP displacement (0.06±0.05s, 0.13±0.05s and 0.67±0.05s) when the AP displacement reached its highest value in either the anterior or posterior direction. For the TR angle, two time ranges (0.10±0.05s and 0.50±0.05s) were chosen for analysis, when the TR angle reached its highest value in either the internal or external direction.

### Data analysis

The AP displacement and TR angle outputs at each of 128 points of a human walking gait cycle were averaged across ten gait cycles for each condition from each cadaveric knee sample. The mean AP displacement and TR angles with 95% confidence limits were calculated for the four intact knees (n = 4) to assess the variability between human knee specimens.

## Results

**Fig 4:**
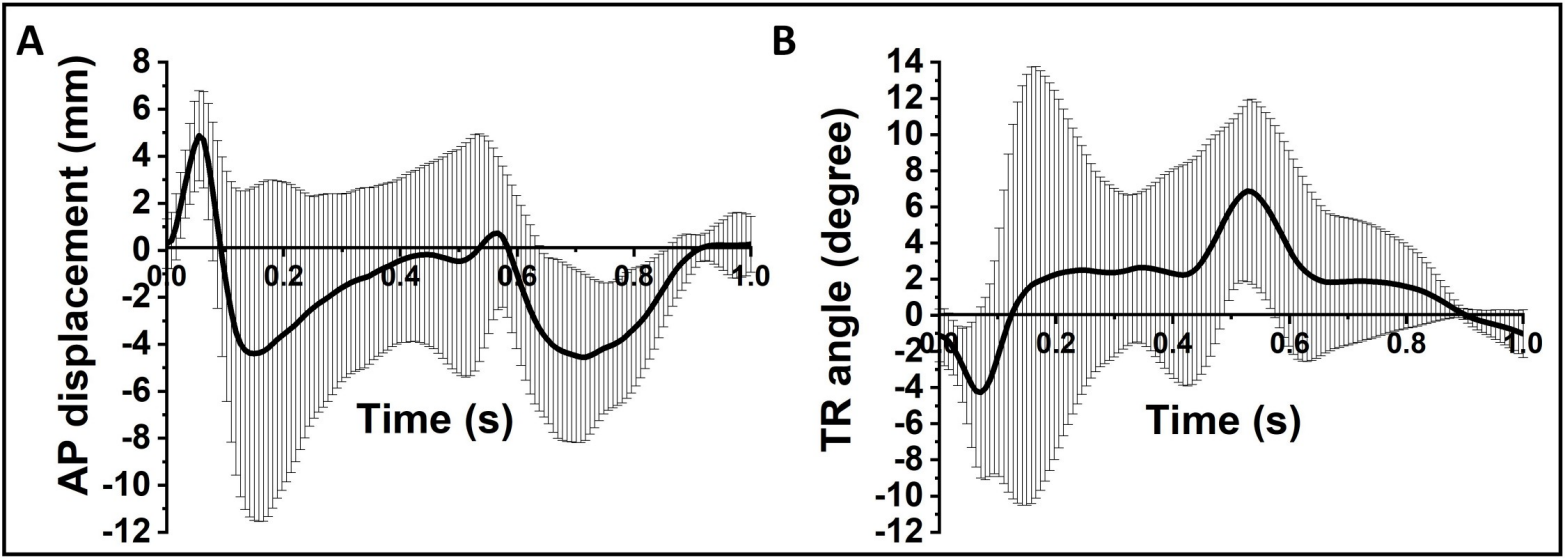
A) Average AP displacement and B) average TR angle of the 4 human knee specimens in a human walking gait cycle (both AP and TR force driven condition). The grey regions indicate 95% confidence intervals of the mean (n = 4) AP displacement and TR angle.

The average AP displacement and TR angle outputs across all four intact cadaveric knees under both AP and TR force driven conditions over a human walking gait cycle are shown in Fig 4. The AP displacement profiles for all four human knee specimens followed closely the shape of the AP force input profile (Fig 2). Three of the human knee specimens demonstrated an additional TR angle peak at the time range of 0.16s-0.3s when the TR torque changed polarity and the FE angle reached the highest flexion. Both AP displacement and TR angle profiles showed large variations among donors as demonstrated by the high 95% confidence limits. The anterior peak value of the AP displacement varied from 2.2 mm to 10.3 mm and the posterior AP peak value varied from 4.1 mm to 6.7 mm. The peak values of the TR angle output varied from 1.0° to 7.0° in the external direction and varied from 4.1° to 11.4° in the internal direction. The results indicated that each individual human knee joint specimen had different knee kinematics as a result of variations in anatomy and soft tissue laxity.

**Fig 5:**
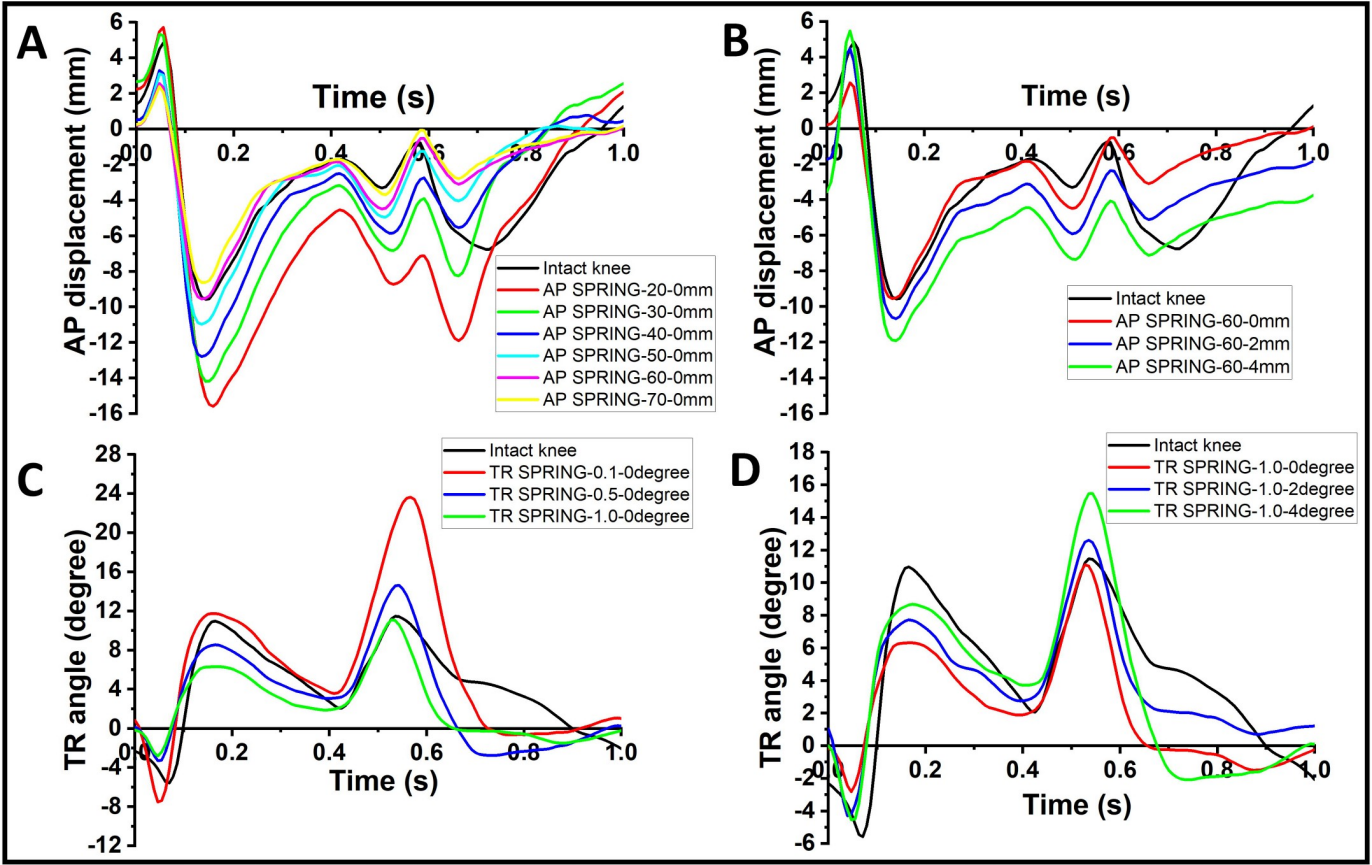
Typical kinematic profiles A) and B) AP displacement; C) and D) TR angle from one donor under different spring conditions for different spring rates (A, C) and free lengths/degrees (B, D).

As shown in Fig 5, the AP displacement and TR angle output profiles under all the spring constraint conditions showed a similar shape to the intact knee, which was observed for all four knees studied. Increasing the AP spring rate from 20 N/mm to 70 N/mm caused incremental decreases in overall AP displacement (Fig 5A) while increasing the free length from 0 mm to 4 mm caused increases in overall AP displacement (Fig 5B), which was consistent with the findings from the porcine knee model [8]. The response of TR angle profiles to spring rate and free angle was similar to that of AP displacement (Fig 5C and 5D).

**Fig 6:**
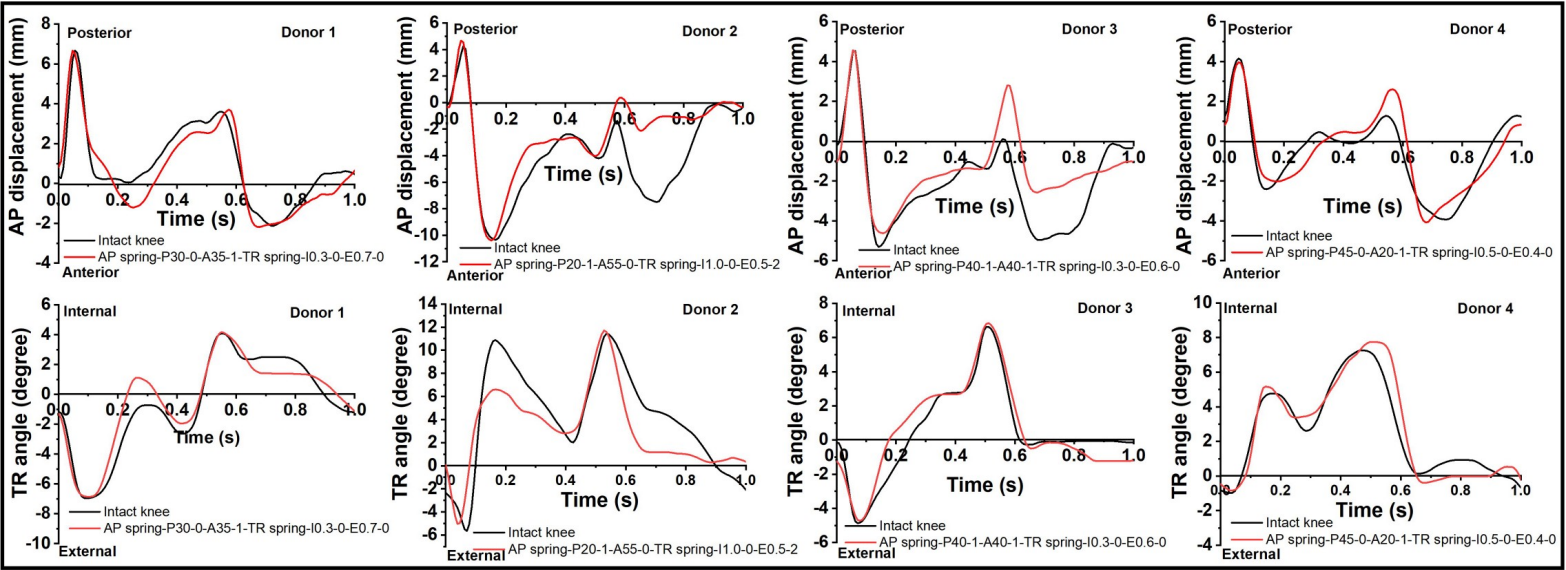
Comparison of kinematic profiles (AP displacement and TR angle) of the intact knee and the most appropriate spring constraints from four human knee specimens.

The resulting AP displacement and TR angle output profiles following selection of the most appropriate spring conditions matched closely those profiles from each individual intact knee (Fig 6). The results showed that the methodologies were successful in finding the most appropriate spring conditions for each individual human knee joint. For donors 1 and 4, the maximum anterior AP displacements appeared in the time range of 0.67±0.05s, while for donors 2 and 3, the maximum anterior AP displacements were in the time range of 0.13±0.05s. Therefore, the time point for comparing the spring conditions with the intact knee was specimen specific according to the time points where the maximum AP displacement occurred. In addition, the maximum values of both AP displacement and TR angle for each knee were different, the deviations of maximum AP and TR were up to 7.2 mm and 8.2°, respectively (Fig 4). Therefore, the most appropriate spring condition for each knee was unique and required specific combinations of spring rate and free length/degree in each direction, which are shown in Tables 1 and 2. The majority of the spring conditions were different amongst donor specimens, only donor 1 and donor 3 had the same internal TR spring condition which was 0.3 Nm/° with 0° free rotation. And the most appropriate AP and TR spring conditions for each donor showed minimal differences compared to the intact knee (less than 0.7 mm for AP and less than 0.7° for TR).

**Table 1:** The most appropriate AP spring condition for each donor specimen.

Spring	Polarity	Donor	Spring rate (N/mm)	Free length (mm)
**AP Spring**	**Posterior**	**Donor 1**	30	0
		**Donor 2**	20	1
		**Donor 3**	40	1
		**Donor 4**	45	0
	**Anterior**	**Donor 1**	35	1
		**Donor 2**	55	0
		**Donor 3**	40	1
		**Donor 4**	20	1

For Donor 1, the most appropriate AP spring condition was posterior spring rate of 30 N/mm with 0 mm free length and anterior spring rate of 35 N/mm with 1 mm free length.

**Table 2:** The most appropriate TR spring conditions for each donor specimen.

Spring	Polarity	Donor	Spring rate (Nm/°)	Free angle (°)
**TR Spring**	**Internal**	**Donor 1**	0.3	0
		**Donor 2**	1.0	0
		**Donor 3**	0.3	0
		**Donor 4**	0.5	0
	**External**	**Donor 1**	0.7	0
		**Donor 2**	0.5	2
		**Donor 3**	0.6	0
		**Donor 4**	0.4	0

For Donor 1, the most appropriate TR spring condition was an internal spring rate of 0.3 Nm/° with 0° free rotation and an external spring rate of 0.7 Nm/° with 0° free rotation.

## Discussion

The aim of this study was to develop an *in vitro* pre-clinical simulation model of the human cadaveric knee with appropriate soft tissue constraints to evaluate the functional performance of early-stage knee soft tissue interventions. Four human cadaveric knees were investigated and the most appropriate soft tissue constraints for each individual knee were determined.

Our previous study [8] demonstrated the effect of AP spring constraints on the kinematic outputs in the porcine knee simulation model, which showed that increasing the free length caused an increase in the AP displacement and increasing the spring rate caused a decrease in the AP displacement. The porcine model also indicated that a hard spring with a higher spring stiffness required a larger free length setting and a soft spring required a smaller free length in order to match the kinematic output of the natural porcine knee. These findings were used to inform this study for investigating the most appropriate spring conditions in both AP and TR directions in the human knee simulation model.

Unlike the porcine knee model, the output kinematic profiles for each intact individual human cadaveric knee under the same input force profile showed large variations, most likely due to the variations in geometry and soft tissue laxity of each human knee joint. The results also showed that each dissected individual human knee displayed different kinematic outputs under the same spring conditions, which was likely due to the differences in the geometry of the dissected human knees. These findings confirmed that the most feasible methodology for developing the human knee model was to adjust the spring conditions (spring rates and free lengths/degrees) to simulate the specific kinematic response from each individual knee.

Virtual springs were used in this study rather than physical springs used in our previous studies [8]. It showed the ability to simulate any response profile by overcoming the option limitation of spring rate and gap from the physical springs. The output kinematic profiles of the resected human knee joint under the virtual spring conditions showed similar shapes to the kinematic profiles of the intact knee, however, similar to the physical spring, the virtual spring was not able to simulate all of the features of the intact knee. For example, for donor 2 and donor 3, the highest anterior AP peak was observed at the time of 0.13±0.05s when it reached the highest anterior AP force, and a second anterior AP peak appeared during the swing phase (0.6s-0.8s) when a smaller anterior AP force was applied. The most appropriate spring condition matched closely to the highest AP peak but showed a lower AP displacement compared to the second AP peak of the intact knee.

The use of simplified springs in two axes to replicate the complex function of soft tissues in the knee is challenging, and indeed the force-displacement and torque-rotation relationships were different between the natural soft tissues and springs. The spring simulation system in the experimental simulator can only represent passive soft tissue constraint forces and not the active forces of the muscle in the knee. This is a limitation of the model but does not mean that the model does not work. Secondly, this study has shown the importance of developing specimen-specific constraints, as the motion of each knee in response to the same kinematic inputs is unique to each individual specimen. The use of virtual spring constraints provided a simple and time-efficient way to simulate the function of the soft tissue in the knee simulator compared to the physical spring, but it was not able to fully replicate the characteristics of the soft tissues throughout the entire gait cycle. Despite this, a process for determining the most appropriate spring conditions which closely replicated the kinematics of the native intact knee as much as possible, taking into consideration the limitations of the simulation, was successfully developed.

The ISO standard for wear testing of total knee replacements [24] recommends the use of springs with a stiffness of 9.3 N/mm and with a ± 2.5 mm gap in the AP direction and a spring of 0.13 Nm/° in the TR direction to simulate the soft tissue function of the natural human knee [21, 22]. DesJardins et al. [16] applied an AP spring of 20 N/mm and TR spring of 0.28 Nm/° into their TKR simulation to simulate soft tissue constraints. However, the biomechanics of knee ligaments are different among individuals [28]. Those spring settings only represent the average population and do not simulate the variation in population groups. Van Houtem et al. investigated two types of AP spring, 7.24 N/mm and 33.8 N/mm, in a cadaveric knee model and suggested that an intermediate stiffness would be more accurate in replicating the AP motion [17]. This current study showed the most appropriate AP spring rate for the four human knees varied from 20 to 55 N/mm and the TR spring rate varied from 0.3 to 1.0 Nm/°.

There are a few reasons that might explain the differences in spring constraints between the studies. Firstly, the intact knee in this study kept the whole knee joint structures including the patella while the patella was sacrificed in the study from van Houtem [17]. A different knee simulator was also used in this study. The current study used a novel single station six-axis electro-mechanical knee simulator that was specifically designed for studying the tribology of a natural knee joint [6]. Previous studies have used simulators designed for the investigation of knee prostheses [16, 17, 21, 22]. Finally, the biological variability in knee geometries and structures could have resulted in different kinematic outputs which in turn required different spring constraints.

Secondly, the majority of studies are based on the findings of Fukubayashi et al. [20], which identified the ligament load-displacement curve in cadaveric knees under static loading conditions. Their results showed smaller variations in kinematic output under static loading conditions compared to the current study which applied dynamic load. This study investigated the soft tissue function under dynamic physiological motions and loading scenarios, which is of greater clinical relevance than static loads at discrete flexion angles. The kinematic outputs measured under dynamic (loading and motion) conditions were affected not only by the stiffness of the ligament and other soft tissue but also the size and geometry of the joint. Biological variation in the donors’ knees played a more important role under dynamic conditions than the static loading conditions, which resulted in the larger variations observed in kinematic outputs in this study.

Furthermore, previous cadaveric studies have shown that kinematic output was also significantly affected by compression load and flexion angles [29–31]. Therefore, it is not possible to use an average spring setting for every knee to simulate the complex response of the AP translation and tibial rotation under dynamic loading conditions and it is difficult to compare data between different studies due to variations in testing conditions.

In any study involving cadaveric human tissue, the sample size is a critical ethical issue. We carefully considered the sample size during the design of this study, and four samples were considered to be adequate for the following reasons.

Firstly, the sample number was decided upon by considering the nature of this study. This was a sample-specific study, to demonstrate the efficacy of the method. The purpose of the study was not to determine whether there was significant variation between samples for any of the testing variables. This is in contrast to other studies, in which 6–10 cadaveric samples have been used to ensure adequate power to detect significant differences in the variable under investigation [32–34].

Secondly, this study was not designed to investigate the effect of biological variables such as gender, age or body mass index on the biomechanical function of the knee. This study developed a sample-specific tool to simulate soft tissue function in each human cadaveric knee joint. In similar types of studies, only one, two or three specimens have been used to test sample-specific tools [14, 15, 35].

Thirdly, the main goal of this simulation model was to develop a methodology to provide a platform to study knee interventions, which was an advance from previous porcine models. The methodology has previously been validated on a sufficient number of porcine samples (n = 6) [8] and a pilot human study was performed before studying the four samples in the current study. This prior work combined with the results from this specimen-specific study represents a large body of work that gives further confidence in the specimen-specific methodology.

This *in vitro* model does have limitations. The input profile applied in the current study was adopted from the ISO standard for wear of total knee-joint prostheses, which is intended to mimic joint contact forces that occur during walking. This does not fully reproduce *in vivo* conditions. Due to the absence of active muscle forces in the cadaveric knee joint, the kinematic outputs from the simulation system might not be representative of *in vivo* mechanics. Furthermore, the *in vivo* joint biomechanics are unique for each individual human knee, which are influenced by factors such as body mass index, alignment and osteoarthritis [36]. Therefore, future work will apply specimen-specific loading profiles to this *in vitro* human knee model to more closely simulate the *in vivo* biomechanics for each individual knee. Future studies will apply this model to assess the functional performance of a variety of early-stage soft tissue repair therapies in the knee, for example, meniscus allografts.

## Conclusions

This study has successfully developed a methodology for using virtual spring constraints to simulate soft tissue function in individual human cadaveric knee joints. It is the first *in vitro* human knee model which can simulate specimen-specific biomechanical function, which will provide a more comprehensive and reliable assessment of novel soft tissue interventions in the knee.
